# Decreased Complexity in Alzheimer's Disease: Resting-State fMRI Evidence of Brain Entropy Mapping

**DOI:** 10.3389/fnagi.2017.00378

**Published:** 2017-11-20

**Authors:** Bin Wang, Yan Niu, Liwen Miao, Rui Cao, Pengfei Yan, Hao Guo, Dandan Li, Yuxiang Guo, Tianyi Yan, Jinglong Wu, Jie Xiang, Hui Zhang

**Affiliations:** ^1^College of Computer Science and Technology, Taiyuan University of Technology, Taiyuan, China; ^2^Department of Radiology, First Hospital of Shanxi Medical University, Taiyuan, China; ^3^School of Life Science, Beijing Institute of Technology, Beijing, China; ^4^Key Laboratory of Convergence Medical Engineering System and Healthcare Technology, Ministry of Industry and Information Technology, Beijing Institute of Technology, Beijing, China; ^5^Key Laboratory of Biomimetic Robots and Systems, Ministry of Education, Beijing Institute of Technology, Beijing, China; ^6^Graduate School of Natural Science and Technology, Okayama University, Okayama, Japan

**Keywords:** Alzheimer's disease, mild cognitive impairment, resting-state functional magnetic resonance imaging, permutation entropy, complexity

## Abstract

Alzheimer's disease (AD) is a frequently observed, irreversible brain function disorder among elderly individuals. Resting-state functional magnetic resonance imaging (rs-fMRI) has been introduced as an alternative approach to assessing brain functional abnormalities in AD patients. However, alterations in the brain rs-fMRI signal complexities in mild cognitive impairment (MCI) and AD patients remain unclear. Here, we described the novel application of permutation entropy (PE) to investigate the abnormal complexity of rs-fMRI signals in MCI and AD patients. The rs-fMRI signals of 30 normal controls (NCs), 33 early MCI (EMCI), 32 late MCI (LMCI), and 29 AD patients were obtained from the Alzheimer's disease Neuroimaging Initiative (ADNI) database. After preprocessing, whole-brain entropy maps of the four groups were extracted and subjected to Gaussian smoothing. We performed a one-way analysis of variance (ANOVA) on the brain entropy maps of the four groups. The results after adjusting for age and sex differences together revealed that the patients with AD exhibited lower complexity than did the MCI and NC controls. We found five clusters that exhibited significant differences and were distributed primarily in the occipital, frontal, and temporal lobes. The average PE of the five clusters exhibited a decreasing trend from MCI to AD. The AD group exhibited the least complexity. Additionally, the average PE of the five clusters was significantly positively correlated with the Mini-Mental State Examination (MMSE) scores and significantly negatively correlated with Functional Assessment Questionnaire (FAQ) scores and global Clinical Dementia Rating (CDR) scores in the patient groups. Significant correlations were also found between the PE and regional homogeneity (ReHo) in the patient groups. These results indicated that declines in PE might be related to changes in regional functional homogeneity in AD. These findings suggested that complexity analyses using PE in rs-fMRI signals can provide important information about the fMRI characteristics of cognitive impairments in MCI and AD.

## Introduction

Alzheimer's disease (AD) is a neurodegenerative disease that is characterized by declines in cognition and memory (Brookmeyer et al., 2007). The neuropathology of AD is characterized by neuronal loss and the appearance of neuritic plaques containing amyloid-β-peptide and neurofibrillary tangles (Haass and Selkoe, 2007). And these changes lead to abnormal brain function in AD (Cho et al., 2013). Mild cognitive impairment (MCI) is an intermediate state between normal aging and AD and is associated with a high risk of progression to AD (Petersen and Negash, 2008). Several neuroimaging techniques, including magnetic resonance imaging (MRI), blood oxygenation level-dependent (BOLD) functional MRI (fMRI), and positron emission tomography (PET), have been explored to study brain function in AD (Lebedeva et al., 2017; Li et al., 2017). Usually, the brain function of AD patients is weakened including problems storing and retrieving information due to the destruction of neurons in parts of the patient's brain. Then, AD affects the brain areas involved in language and reasoning. Eventually, the most notable characteristic of AD on MRI is cerebral atrophy in the medial temporal lobe, hippocampus, right temporal lobe, precuneus, cingulate gyrus, and inferior frontal cortex (Möller et al., 2013). The changes in these brain regions suggest that they are relevant for the loss of functionality in patients with dementia. A differential diagnosis for the types of dementia should be attempted. Recently, resting-state fMRI (rs-fMRI) has been introduced as an alternative approach for studying brain functional abnormalities in AD (Gusnard and Raichle, 2001; Fox and Raichle, 2007).

Based on its structure and function, the human brain is one of the most complex information processing systems. Complexity can be defined as the difficulties that arise when describing or predicting a signal. Normal physiology requires a complex network to effectively control function. Lipsitz and Goldberger (Lipsitz, 1992, 2004) argued that with aging and disease, losses of complexity occur in the dynamics of many integrated physiological processes of an organism. The development of the concept of complexity has focused on measuring regularity using various metrics that are based on non-linear time series analysis. Lyapunov exponents (Wolf et al., 1985) and correlation dimensions (Broock et al., 1996) have been used to characterize non-linear dynamics, but they require large data sets (Eckmann and Ruelle, 1992) and assume that the time series is stationary (Grassberger and Procaccia, 1983), which is typically inappropriate for biological data. Entropy measures the randomness and predictability of a stochastic process and generally increases with greater randomness; i.e., lower entropy indicates lower signal complexity.

Previous research has noted decreased complexity in the EEG and MEG signals of aging and diseased brains. Gomez et al. used approximate entropy (ApEn) and sample entropy (SampEn) to analyze MEG signals and found that the signals were less complex and more regular in AD patients than in control subjects (Gómez and Hornero, 2009; Gomez et al., 2010). Recent EEG studies have explored event-related multiscale entropy (MSE) measures as features for effectively discriminating between normal aging, MCI, and AD and found decreasing complexity with the severity of cognitive decline (McBride et al., 2014a,b). These findings indicate decreases in the EEG and MEG signal complexities of AD and MCI patients.

Entropy is a commonly used metric for the measurement of brain complexity. Permutation entropy (PE) is a new method that is used to measure the irregularity of non-stationary time series (Bandt and Pompe, 2002). PE considers only the ranks of the samples and not their metrics. As an ordinal measure, PE has some advantages over other commonly used entropy measures, such as ApEn (Pincus, 1991) and SampEn (Richman and Moorman, 2000), including its simplicity, low complexity in computation without further model assumptions, and robustness in the presence of observational and dynamical noise (Zanin et al., 2012). PE has been used in EEG signal studies of human absence epilepsy (Ferlazzo et al., 2014), typical absences (Li et al., 2014), MCI (Timothy et al., 2014), and AD (Morabito et al., 2012). These studies suggest that PE is a useful tool for the study of abnormalities of brain complexity.

Few studies have performed complexity analyses of rs-fMRI signals (Liu et al., 2013; Sokunbi et al., 2013). To the best of our knowledge, PE has not been applied to the complexity study of rs-fMRI signals. Some fMRI studies have found that complexity decreases in gray and white matter and some brain regions with normal aging (Liu et al., 2013; Sokunbi et al., 2015). Compared with normal controls (NCs), AD patients exhibit a greater decrease in cognitive ability and memory. EEG studies have demonstrated that declining brain function is associated with decreased complexity in the brains of AD patients (McBride et al., 2014a,b). Liu et al. found that cognitive impairment was associated with decreased complexity of the fMRI signals in the gray matter and brain regions in a familial AD group (Liu et al., 2013). However, the alterations in the complexities of rs-fMRI signals in MCI and AD patients remain unclear.

In the present study, an analysis of PE complexity was performed using rs-fMRI signals of NC, early MCI (EMCI), late MCI (LMCI), and AD subjects from the Alzheimer's disease neuroimaging initiative (ADNI, http://adni.loni.usc.edu/) database. First, PE brain maps of the four groups were extracted and subjected to Gaussian smoothing. One-way analysis of variance (ANOVA) was performed to identify the significantly different clusters. Then, the average PEs of the selected regions of interest (ROIs) were analyzed. Finally, Pearson's correlations between the average PEs of ROIs for each participant and each of the Mini-Mental State Examination (MMSE), Functional Assessment Questionnaire (FAQ) and global Clinical Dementia Rating (CDR) scores were analyzed. Moreover, we examined the relationships between regional homogeneity (ReHo) and PE in AD and MCI patients. ReHo is suitable for exploring resting-state functional homogeneity (Zang et al., 2004). A larger ReHo value indicates higher regional synchronization. We also examined the relationships between glucose metabolism on FDG-PET and PE in AD and MCI patients. Finally, we examined the gray matter volumes in the four groups using a voxel-based morphometry (VBM) method and studied the relationship between the gray matter volumes and PEs in the patient groups.

The objective of our study was to determine the alterations of complexity in MCI and AD patients from brain entropy maps based on rs-fMRI data. We also found that these alterations were related to the changes in regional synchronization present in MCI and AD compared with NCs.

## Materials and methods

### Participants

All of the subjects were selected from the ADNI (ADNI-2) database. The ADNI aims to study the pathogenesis and prevention of AD by analyzing various medical imaging data.

A total of 124 subjects were selected, and the data from each subject consisted of 140 functional volumes from the database according to disease type (AD, LMCI, EMCI, and NC). The subjects included 29 AD patients (average age of 72.33 years, 18 females), 32 LMCI patients (average age of 72.57 years, 13 females), 33 EMCI patients (average age of 72.01 years, 16 females), and 30 NC subjects (average age of 74.18 years, 19 females; Table 1). The AD patients had MMSE scores of 14–26, the LMCI patients had MMSE scores of 23–28, the EMCI patients had MMSE scores of 24–30, and the NC subjects, who did not exhibit depression or dementia, had MMSE scores of 24–30. The FAQ is a measure of the ability to perform 10 high-level skills used in daily tasks (shopping, preparing meals, handling finances, and understanding current events), each of which is rated by a knowledgeable informant. The total score ranges from 0 to 50, and higher scores indicating poorer functional performance. The AD patients had FAQ scores of 3–28, the LMCI patients had FAQ scores of 0–18, the EMCI patients had FAQ scores of 0–12, and the NC subjects had FAQ scores of 0–3. The global CDR scores are discrete values of 0, 0.5, and 1 that indicate no dementia, mild dementia, and dementia, respectively. All AD patients had a global CDR of 0.5 or 1, the LMCI and EMCI patients had global CDR scores of 0.5, and the NC subjects had a global CDR score of 0.

**Table 1:** Demographic and clinical information of the participants.

**Group**	**NC**	**EMCI**	**LMCI**	**AD**	***P*-value**
Age (years)^a^	74.18 ± 5.96	72.01 ± 5.87	72.57 ± 8.16	72.33 ± 7.26	0.505
Sex (M/F)	11/19	17/16	19/13	11/18	0.732
MMSE^b^	28.9 ± 1.7	27.59 ± 2.02	26.96 ± 2.69	21.0 ± 3.5	< 0.001
FAQ^c^	0.14 ± 0.44	3.03 ± 4.50	4.07 ± 4.70	15 ± 7.47	< 0.001
CDR^d^	0	0.5	0.5	0.84 ± 0.23	< 0.001

a, b, c, d *Values represent the mean ± standard deviation*.

*MMSE, Mini-Mental State Examination; FAQ, Functional Assessment Questionnaire; CDR, Clinical Dementia Rating*.

### Data acquisition

All subjects were scanned in a three-tesla (3T) scanner. During the resting-state scans, the subjects were asked to keep their eyes closed (Jack et al., 2008). Functional and structural MRI data were collected with the following parameters: field strength = 3.0; manufacturer = Philips Medical Systems; slice thickness = 3.3; repetition time (TR) = 3,000 ms; echo time (TE) = 30 ms; flip angle = 80°; and slice number = 48.

FDG-PET images were acquired at a variety of scanners nationwide using either a 30-min six-frame scan or a static 30-min single-frame scan acquired 30–60 min post-injection (details are available at https://adni.loni.usc.edu/wp-content/uploads/2010/05/ADNI2_PET_Tech_Manual_0142011.pdf).

### Data preprocessing

The preprocessing of rs-fMRI data was performed using the Data Processing Assistant for Resting-State fMRI (DPARSF) toolbox (Chao-Gan and Yu-Feng, 2010) and the SPM8 package (http://www.fil.ion.ucl.ac.uk/spm). Briefly, the preprocessing steps were as follows: the first 10 volumes of the functional images during the participant's adaptation to the circumstances were discarded; slice-timing correction was performed according to the last slice; the images were realigned for head movement compensation using a six-parameter rigid-body spatial transformation because excessive head motion may induce large artifacts in fMRI time series; the images were normalized to the Montreal Neurological Institute (MNI) space; and finally, the signal drift was removed using a linear model. Additionally, spatial smoothing of the brain PE maps was performed to reduce the white noise and suppress the effects due to residual differences during inter-subject averaging using an 8-mm full-width at half maximum (FWHM) smoothing kernel (Sokunbi et al., 2015). Notably, PE complexity was accomplished by voxel-based analysis to explore regional differences, and smoothing before a PE calculation will greatly increase the regional similarity (Chao-Gan and Yu-Feng, 2010). A recent study involving fuzzy approximate entropy analysis of rs-fMRI signals performed the smoothing after the entropy calculation (Sokunbi et al., 2015). Moreover, the ReHo explores the functional homogeneity of resting-state fMRI data (Zang et al., 2004), which might provide convenience in the potential explanation of PE. Thus, we calculated the ReHo after preprocessing. Then, the ReHo of the brain was smoothed with an 8-mm FWHM smoothing kernel.

Some studies have shown that the removal of nuisance signals had influence on the results (Chao-Gan and Yu-Feng, 2010; Wang et al., 2014). We also tried to remove the effect of nuisance covariates, including the global signal, the motion parameters, the cerebrospinal fluid (CSF), and the white matter signals. The detailed data processing, statistical analyses, and results were presented in Presentation 1 (Supplementary Material).

The analysis of the gray matter volume was performed according to the VBM protocol using DPARSF (Chao-Gan and Yu-Feng, 2010). This process primarily consisted of segmentation and normalization. First, each subject's MRI data were segmented into gray matter, white matter and cerebrospinal fluid (CSF). Subsequently, diffeomorphic anatomical registration using exponential lie algebra (DARTEL) was applied to normalize the gray matter images and iteratively create the template. The subjects' gray matter images were registered to new templates for each iteration. Then, the normalized gray matter images were multiplied to preserve the absolute volume of the gray matter in the subjects' native spaces. Finally, all gray matter images were smoothed with an 8-mm FWHM Gaussian kernel.

Preprocessing of the FDG-PET scans was performed using the SPM8 package (http://www.fil.ion.ucl.ac.uk/spm). Dynamic scans were registered to the mean frame and averaged to create a single average image. Then, the images were normalized to the MNI space (voxel size: 3 × 3 × 3). Next, spatial smoothing was performed using a Gaussian smoothing kernel with FWHM of [8 8 8]. Therefore, each voxel time series was standardized to a mean of zero and a standard deviation of unity to allow the data sets to be compared. The scans were intensity-normalized using a whole-cerebellum reference region to create standardized uptake value ratio (SUVR) images.

### PE algorithm

The basic principle of PE is that it does not consider the specific values of the data; rather, PE is based on the comparison of adjacent data points in the time domain. The algorithm is described below.

Given a time series *x*(*i*), *i* = 1, 2, ….., *N*, a vector composed of the m-th subsequent values is constructed as follows:

(1) $$\begin{array}{c} X(1)=\{x(1),x(1+l),\cdots ,x(1+(m-1)l)\}\\ \vdots \\ X(i)=\{x(i),x(i+l),\cdots ,x(i+(m-1)l)\}\\ \vdots \\ X(N-(m-1)l)=\{x(N-(m-1)l),\\ x(N-(m-2)l),\cdots ,x(N)\} \end{array}\}$$

where *m* is the embedding dimension, and *l* is the delay time.

The vector *X*(*i*) can be rearranged in an ascending order as follows:

(2) $$\begin{array}{l} X(i)=\{x(i+(j_{1}-1)l)\leq x(i+(j_{2}-1)l)\leq \cdots \leq \\ x(i+(j_{m}-1)l) \end{array}$$

where *j* = 1, 2, ⋯, *m*. Note that if two values are equal (here, *x*(*i* + (*j*_1_ − 1)*l*) = *x*(*i* + (*j*_2_ − 1)*l*)), they are ordered according to the size of the *j*_1_, *j*_2_ value, such that *x*(*i*+(*j*_1_ − 1)*l*) ≤ *x*(*i*+(*j*_2_ − 1)*l*) when *j*_1_ < *j*_2_. Then we can obtain a set of symbol sequences by each raw of it that the reconstructed matrix of any time series, where the symbol sequences just like *S*(*g*) = {*j*_1_, *j*_2_, ⋯, *j*_*m*_}, (*g* = 1, 2, ⋯*k, k* ≤ *m*!), where the *k* means the objectively quantity of {*j*_1_, *j*_2_, ⋯, *j*_*m*_}. So, any vector *X*(*i*) is uniquely mapped into (1, 2, ⋯, *m*) or (2, 1, ⋯, *m*)⋯ or (*m, m* − 1, ⋯, 1) in total *m*! possible symbol sequences and *S*(*g*) is one of them. Then, let the probability distribution of the distinct symbols be *P*_*g*_(*g* = 1, 2, ⋯*k*). The PE is defined as the Shannon entropy for the *k* distinct symbols:

(3) $$PE=-\sum_{g=1}^{k}P_{g}\ln P_{g}$$

Be aware, PE reaches its maximum ln (*m*!) when *p*_*g*_ = 1/*m*!. Therefore, PE is standardized by ln (*m*!):

(4) $$PE_{s}=PE/\ln(m!)$$

Obviously, the range of *PE*_*s*_ is 0 ≤ *PE*_*s*_ ≤ 1.

*PE*_*s*_ is the local order structure of the time series. A large PE value indicates a more random time series, whereas a small PE value indicates that the time series is regular.

### Computation of PE

In the calculation of PE, three parameter values must be considered and set, including the length of the time series *N*, the embedding dimension *m* and the time delay *l*. Bandt et al. (Bandt and Pompe, 2002) suggested that the embedding dimension should range from 3 to 7 because if the value is too small, the reconstructed sequence contains too few states; therefore, the algorithm loses its meaning and validity and cannot detect the dynamic mutation of the time series. However, if the value is too large, the phase space reconstruction will homogenize the time series, the calculation will be time consuming, and subtle changes in the sequence will not be reflected. The time delay *l* has little influence on the entropy of the time series (Mateos et al., 2014). To allow every possible order pattern of dimension *m* to occur in a time series of length *N*, the condition *m*! ≤ *N* − (*m* − 1)*l* must hold. Moreover, to avoid undersampling, *N* ≥ *m*! + (*m* − 1)*l* is required. Therefore, we need to choose *N* ≥ (*m* + 1)!. For *N* = 130, an obviously unsatisfying complexity estimation is obtained when *m* ≥ 5. To satisfy this condition, we therefore chose a low dimension, i.e., *m* = 4, when calculating the permutation entropy. In the present study, we chose *m* = 4, *l* = 1 for calculation and analysis (Li et al., 2014).

### Statistical analyses

The first statistical tests were performed using the rs-fMRI Data Analysis Toolkit (REST 1.8) (Song et al., 2011). One-way ANOVA was performed to examine differences among the four groups (NC, EMCI, LMCI, and AD). Clusters that were significantly different after adjusting for age and sex differences were selected by setting *P* < 0.005 with a Gaussian random fields (GRF) correction.

The DPARSF toolbox was used to define the ROIs to extract the average PE, ReHo, and PDG-PET values according to the peak MNI coordinates (XYZ), and the radius of the spheres was 8 mm.

The subsequent statistical tests were performed using Statistical Package for Social Sciences (SPSS 20.0; New York, NY, USA) software. The averages PEs of the ROIs of each subject were obtained and one-way ANOVA was performed to examine the differences among the four groups. The relationships between the PE and the clinical measurements of MMSE, FAQ, and CDR were analyzed using Pearson's correlations in the patient groups.

Pearson's correlation analyses of the PE with the ReHo and FDG-PET data were performed in the patient groups using SPSS. Moreover, we also performed correlation analyses between the PEs and the gray matter volumes in the patient groups.

## Results

### Demographic and clinical data

The demographic and clinical data for each group were summarized in Table 1. The means (±*SD*) were presented for the baseline clinical tests. The results of one-way ANOVAs revealed significant effects of group on the MMSE (*F* = 40.924, *P* < 0.001), FAQ (*F* = 61.810, *P* < 0.001), and the CDR (*F* = 238.31, *P* < 0.001) scores but not sex (*F* = 0.431; *P* = 0.732) or age (*F* = 0.785; *P* = 0.505). Note that a higher FAQ score represents greater impairment, whereas a lower MMSE represents greater impairment. The MMSE scores were significantly lower in the MCI (*t* = −3.302, *P* = 0.002) and AD (*t* = −9.333, *P* < 0.001) groups than in the NC group. The FAQ scores were significantly higher in the MCI (*t* = 5.642, *P* < 0.001) and AD groups (*t* = 12.625, *P* < 0.001) than in the NC group. The CDR scores were significantly higher in the MCI (*t* = 7.687, *P* < 0.001) and AD groups (*t* = 28.436, *P* < 0.001) than in the NC group.

### rs-fMRI PE brain maps

**Figure 1:**
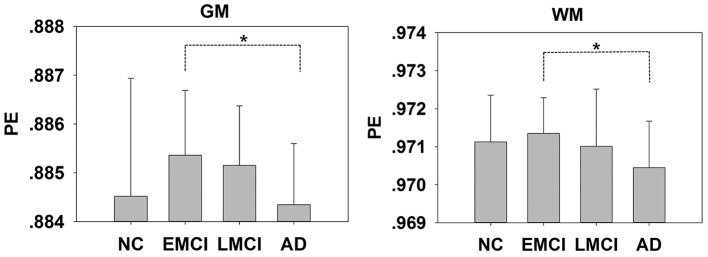
Mean PE values of the gray matter (GM) and white matter (WM) in the NC, EMCI, LMCI, and AD subjects. Significant differences between pairs of groups after Bonferroni correction (*P* < 0.05) are indicated. ^*^P < 0.05. The error bars indicate the *SD*s.

**Figure 2:**
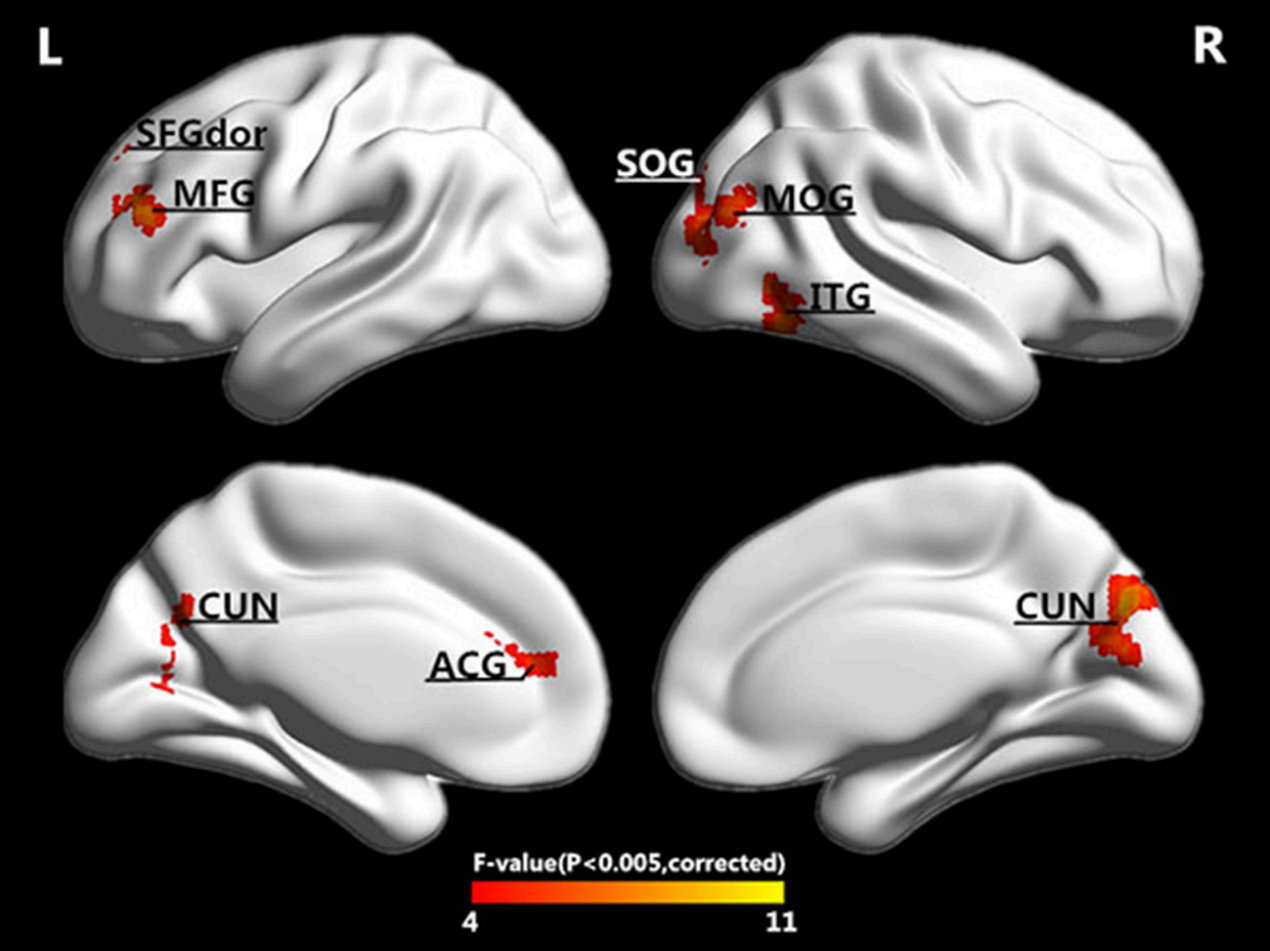
Surface-rendered images showed the differences between the control and patient groups after adjusting for age and sex. The regions showed exhibited different complexities among the four groups. See Table 2 for a complete list of these regions (threshold *P* < 0.005, GRF corrected).

We extracted the mean PEs of the whole brain, gray matter (GM), white matter (WM), and cerebral spinal fluid (CSF). There were differences in the GM (*F* = 2.711, *P* = 0.048) and WM (*F* = 2.792, *P* = 0.043) but no differences in the whole brain (*F* = 1.713, *P* = 0.168) or CSF (*F* = 1.183, *P* = 0.319) among the four groups. The results of the one-way ANOVAs were presented in Figure 1. At the regional levels, five clusters were found to exhibit significant differences in PE among the four groups, as illustrated in Figure 2. The complexity differences among the four groups were mainly observed in the temporal, occipital, and frontal lobes. The results after removing the effect of nuisance covariates showed the complexity differences were mainly observed in the frontal lobes (Presentation 1 in Supplementary Material).

### ROI analysis

**Figure 3:**
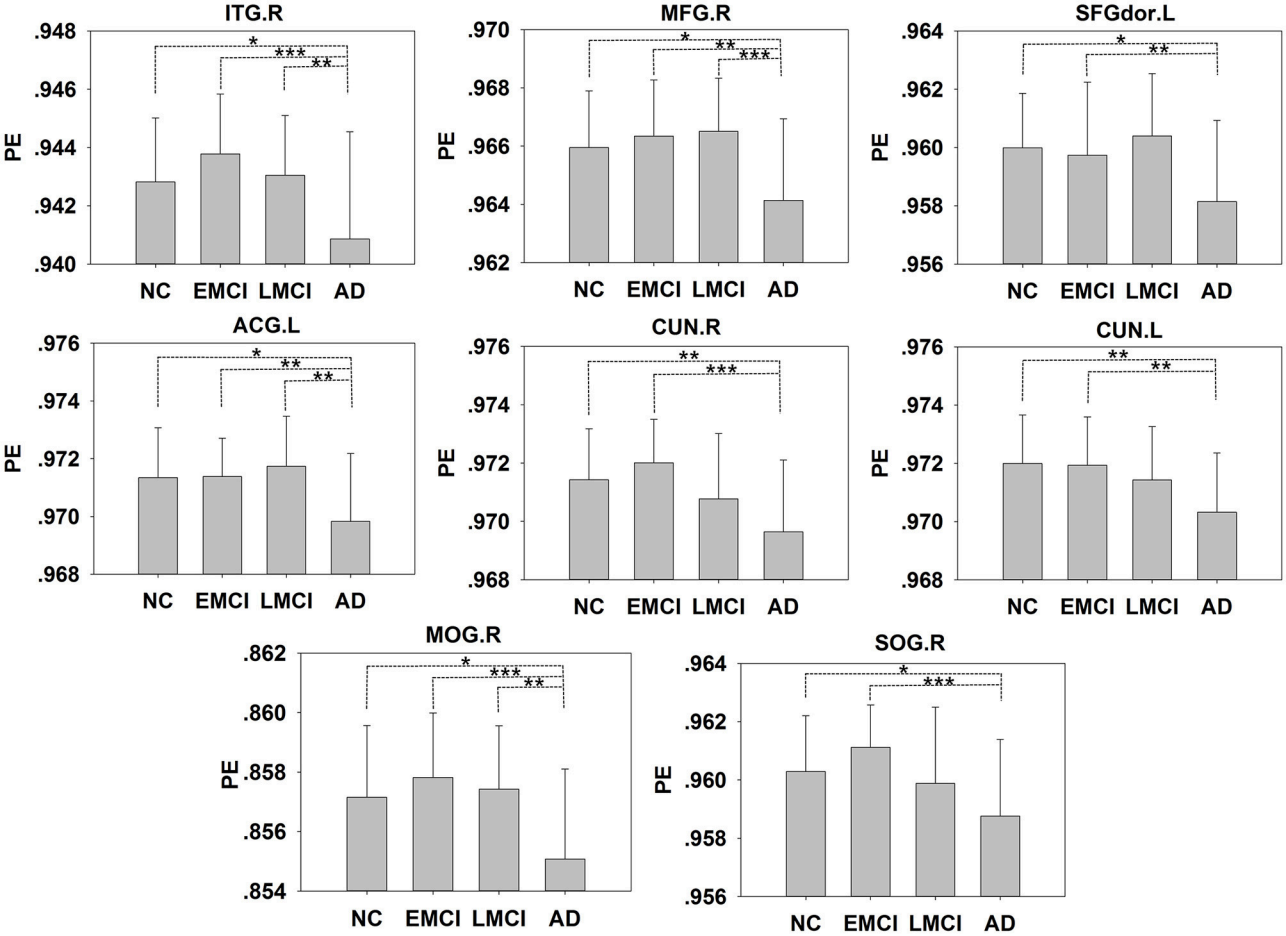
The PE values of the NC, EMCI, LMCI, and AD subjects. Significant differences between pairs of groups after Bonferroni correction (*P* < 0.05) are indicated. ^*^P < 0.05, ^**^P < 0.01, ^***^P < 0.001. The error bars indicate the *SD*s.

In addition to the brain regions at the peak points, other significant brain regions that accounted for a large proportion were also extracted. We obtained eight ROIs from five clusters for the next analysis as presented in Table 2. The average PE values were extracted according to the peak MNI coordinates of the ROIs, and the sphere radius was 8 mm. Specifically, as presented in Table 2, the following regions exhibited significant differences: the right inferior temporal gyrus (ITG.R), the left middle frontal gyrus (MFG.L), the left superior frontal gyrus (SFGdor.L), the left anterior cingulate and paracingulate gyri (ACG.L), the right cuneus (CUN.R) and left cuneus (CUN.L), the right middle occipital gyrus (MOG.R), and the right superior occipital gyrus (SOG.R). In particular, five peak MNI coordinate regions (ITG.R, MFG.L, ACG.L, CUN.R, and MOG.R) exhibited statistically significant differences (*F* > 8.13, *p* < 0.005, corrected). The results of one-way ANOVAs revealed significant effects of group in eight brain regions that exhibited significantly decreased complexity in the AD group compared with the MCI groups and the NC group (*t* > 2.909, *P* < 0.01). Compared with the NC, decreased complexity was also found in the left cuneus in the MCI group (*P* = 0.04, one-tailed uncorrected). Figure 3 illustrated that the PE values were lowest in the AD patients in all of the clusters. Specifically, four brain regions (ITG.R, MFG.L, ACG.L, and MOG.R) exhibited significant differences between the AD patients and the other groups. After removing the effect of nuisance covariates, five clusters were also found decreased complexity in the AD group compared with the MCI groups and the NC group (Presentation 1 in Supplementary Material).

**Table 2:** Characteristics of the brain regions that were significantly different among the four groups.

**Brain region**	**AAL.Abbr**	**Peak MNI (X, Y, Z)**	**Cluster voxels**	**Voxel F value**
Inferior temporal gyrus	ITG.R	(51, 63, 15)	117	8.15
Middle frontal gyrus	MFG.L	(−33, 41, 24)	278	10.82
Superior frontal gyrus	SFGdor.L			
Anterior cingulate gyrus	ACG.L	(−12, 44, 15)	59	8.13
Right cuneus	CUN.R	(12, 78, 30)	126	8.61
Left cuneus	CUN.L			
Middle occipital gyrus	MOG.R	(45, 78, 21)	201	8.42
Superior occipital gyrus	SOG.R			

*The location coordinates are those of the peak significance in each region (P < 0.005, GRF corrected)*.

### Relationships between PE and clinical measurements

The MMSE score is the most widely used brief screening measure of cognition. First, we performed correlation analyses of the MMSE scores with the mean PEs of the whole brain, WM, GM, and CSF in the patient groups (EMCI+LMCI+AD) and found that only the GM (*r* = 0.227, *P* = 0.032) and WM (*r* = 0.210, *P* = 0.049) PEs exhibited positive correlations. We also examined the correlations of the MMSE scores with the PEs of the eight ROIs in the pooled patient groups (EMCI+LMCI+AD). The results were presented in Table 3. The eight ROIs exhibited significant positive correlations between the PEs and MMSE scores (*r* > 0.212, *P* < 0.046) with the MFG.L and MOG.R showing strong positive correlations (*r* > 0.414, *P* < 0.001). A higher MMSE score indicates higher cognitive ability.

**Table 3:** Results of the correlation analyses between the PE maps and the MMSE, FAQ, and CDR scores in the patient groups (EMCI+LMCI+AD).

**Brain region Abbr**.	**MMSE (*r, P*)**	**FAQ (*r, P*)**	**CDR(*r, P*)**
ITG.R	0.312, 0.003^**^	−0.223, 0.036^*^	−0.259, 0.014^*^
MFG.L	0.429, < 0.001^***^	−0.290, 0.006^**^	−0.345, 0.001^***^
SFGdor.L	0.294, 0.005^**^	−0.337, 0.001^***^	−0.365, < 0.001^***^
ACG.L	0.317, 0.002^**^	−0.216, 0.042^*^	−0.355, 0.001^***^
CUN.R	0.349, 0.001^***^	−0.213, 0.045^*^	−0.144, 0.177
CUN.L	0.212, 0.046^*^	−0.125, 0.243	−0.120, 0.262
MOG.R	0.414, < 0.001^***^	−0.326, 0.002^**^	−0.339, 0.001^**^
SOG.R	0.349, 0.001^***^	−0.288, 0.006^**^	−0.211, 0.047^*^
GM	0.227, 0.032^*^	−0.139, 0.193	−0.159, 0.137
WM	0.210, 0.049^*^	−0.112, 0.298	−0.111, 0.302

In the table, r is the Pearson correlation coefficient, and P indicates the level of statistical significance.

* P < 0.05,

** P < 0.01,

*** *P < 0.001. GM, Gray Matter; WM, White Matter*.

The FAQ is more closely tied to functionally relevant abilities, such as accomplishing everyday tasks required for independent living. There were no correlations of the FAQ scores with the mean PEs of the whole brain, WM, GM, or CSF in patient groups. The PEs of seven ROIs exhibited strong negative correlations with the FAQ scores (*r* < −0.213, *P* < 0.045), whereas the CUN.L ROI did not (Table 3). A higher FAQ score indicates poorer functional performance.

The CDR has been validated neuropathologically particularly in terms of the presence or absence of dementia. There were no correlations of the CDR scores with the mean PE of the whole brain or the PEs of the WM, GM, or CSF in the patient groups. The PEs of six ROIs exhibited strong negative correlations with the CDR scores (*r* < −0.211, *P* < 0.047), whereas the PEs of the CUN.L and CUN.R did not (Table 3). A higher CDR score indicates the presence of dementia.

In addition, the correlation analyses were performed between the PE and the clinical measurements in the four groups and consistent significant correlations were found (Table S1). We also found the significant correlations between the PE and the clinical measurements in the patient groups and in the four groups after removing the effect of nuisance covariates (Presentation 1 in Supplementary Material).

### Relationships between PE and ReHo

We extracted the ReHos of 8 ROIs according to the peak MNI coordinates (Table 2), and the sphere radius was 8 mm. We explored the relationship between PE and ReHo in the pooled groups (EMCI+LMCI+AD). The results were presented in Table 4. The GM (*r* = −0.347, *P* = 0.001), WM (*r* = −0.537, *P* < 0.001) and three ROIs (ITG.R, MFG.L, and MOG.R) exhibited significant negative correlations between the PE and ReHo in the patients groups. And the results after removing the effect of nuisance covariates showed that the inferior and middle frontal gyrus exhibited negative correlations between the PE and ReHo in the patient groups (Presentation 1 in Supplementary Material). The results illustrated that high regional spontaneous activities may be associated with a decrease in complexity.

**Table 4:** Results of the correlation analyses between the PE maps and the ReHo, gray matter volume, and FDG-PET values in the patient groups (EMCI+LMCI+AD).

**Brain region Abbr**.	**ReHo (*r, P*)**	**GMV (*r, P*)**	**FDG-PET (*r, P*)**
ITG.R	−0.414, < 0.001^***^	−0.020, 0.845	0.273, 0.019^*^
MFG.L	−0.369, < 0.001^***^	0.081, 0.440	0.027, 0.819
SFGdor.L	−0.179, 0.084	0.074, 0.480	0.043, 0.716
ACG.L	−0.012, 0.909	0.089, 0.391	0.096, 0.417
CUN.R	−0.032, 0.757	0.046, 0.661	0.009, 0.939
CUN.L	−0.020, 0.847	−0.141, 0.175	0.113, 0.341
MOG.R	−0.195, 0.049^*^	0.040, 0.705	0.419, < 0.001^***^
SOG.R	−0.068, 0.515	0.200, 0.053^*^	0.118, 0.320
GM	−0.347, 0.001^***^	0.039, 0.736	0.092, 0.438
WM	−0.537, < 0.001^***^	–	0.078, 0.509

In the table, r is the Pearson correlation coefficient, and P indicates the level of statistical significance.

* P < 0.05,

*** *P < 0.001. GMV, Gray Matter Volume; GM, Gray Matter; WM, White Matter*.

Correlation analyses in the four groups (NC+EMCI+LMCI+AD) were also performed between the PE and ReHo (Table S2). Consistent significant correlations were found.

### Relationships between PE and the gray matter volume, FDG-PET

We extracted the gray matter volumes of eight ROIs according to the peak MNI coordinates, and the sphere radius was 8 mm. Then, we explored the relationships between the PEs and the gray matter volumes in the patient groups. The results were presented in Table 4. The SOG.R exhibited a positive correlation (*r* = 0.200, *P* = 0.053) between the PE and the gray matter volume in the patient groups. Correlation analyses in the four groups (NC+EMCI+LMCI+AD) were also performed between the PE and gray matter volume, and the SOG.R exhibited a significant positive correlation (*r* = 0.210, *P* = 0.010) between the PE and the gray matter volume (Table S2). And the right middle frontal gyrus exhibited a positive correlation (*r* = 0.270, *P* = 0.008) between the PE and the gray matter volume after removing the effect of nuisance covariates (Presentation 1 in Supplementary Material).

Finally, the FDG-PET data of the eight ROIs from the same group of subjects were extracted. Pearson's correlation analyses of the PE and FDG-PET data were performed in the pooled groups (EMCI+LMCI+AD). Two significant correlations were detected (Table 4). The MOG.R (*r* = 0.419, *P* < 0.001) and ITG.R (*r* = 0.273, *P* = 0.019) exhibited significant positive correlations between the PE and FDG-PET data. The correlation analyses in the four groups (NC+EMCI+LMCI+AD) produced consistent results (Table S2).

## Discussion

This study reported the global and regional differences in PE between patients and controls. The significant differences were mainly distributed in the occipital, frontal, and temporal lobes. In the ROI analysis, the AD patients exhibited significantly lower values (lower complexities) than the healthy controls and MCI groups. To identify the continuous distribution of the AD symptoms, we conducted correlation analyses of the PE values and the clinical MMSE, FAQ and CDR scores, all of which revealed an increasing symptom load with decreasing brain activity complexity. We also extracted the regional homogeneities (ReHos) of eight ROIs and performed correlation analyses between the PEs and ReHos, and significant correlations were observed. Additionally, we extracted the FDG-PET data from eight ROIs and performed correlation analyses between the PE and the FDG-PET data. Significant positive correlations between the PE and FDG-PET data were observed in the ITG.R and MOG.R in the patient groups. A positive correlation was found between the PE and the gray matter volume in the patient groups. To summarize, we found significantly decreased complexity in the AD patients, and the results were related to the results of the ReHo analysis.

### Applications of PE for analyzing the complexity of neural signals in the brain

The PE method measures the irregularity of non-stationary time series, and there have been a number of practical applications of complexity measures using EEG data (Li et al., 2007, 2014; Bruzzo et al., 2008). A study demonstrated that the PE can track the dynamical changes of EEG data (Li et al., 2007). Li et al. utilized PE to predict the changes in EEG signals during absence seizures and provided evidence that the three different seizure phases in absence epilepsy can be effectively distinguished (Li et al., 2014). Mammone et al. evaluated PE data extracted from different electrodes in patients with typical absences and healthy subjects (Mammone et al., 2012). Another study used PE as a feature for effectively discriminating between normal aging, MCI, and AD participants (McBride et al., 2014a). In this study, using the PE method, we found decreased complexity in the MCI and AD patients. These findings demonstrated that PE can measure the complexity of neural signals in the brain and disclose abnormalities of the brain in disease states.

In addition to PE, other entropy methods have been used to explore the complexity of fMRI signals. For example, Liu et al. explored the complexity of normal aging using approximate entropy and found that gray and white matter decreased in complexity with normal aging (Liu et al., 2013). Sokunbi MO and co-workers applied approximate entropy, sample entropy, and fuzzy entropy to complexity analyses of the fMRI data in diseases (i.e., schizophrenia and ADHD) and found alterations in complexity compared with normal people (Sokunbi et al., 2013, 2014). We found significantly decreased complexity in MCI and AD patients using the PE analysis that revealed increasing symptom load with decreasing complexity of the brain. We also applied these entropy methods to ADNI datasets, but the differences among the four groups were not significant (results not shown). Compared with other entropy methods, we thought the PE method had the advantages of placing a continuous time series into a symbolic sequence, entailing a faster calculation speed and being more accurate for complexity estimations (Unakafova et al., 2013). Therefore, PE seems to be a useful tool for identifying abnormalities in brain function.

### Decreased complexity in AD

From our results, decreases in complexity were associated with AD. We found decreased complexity in the mean whole-brain PEs of the gray matter and white matter in AD compared with EMCI (Figure 1). One study of the complexity of rs-fMRI data found cognitive impairment was associated with decreases in the gray matter of a familial AD group (Liu et al., 2013). At the regional level, five clusters were found to exhibit significantly decreased complexity in the AD group compared with the MCI and NC groups, and these clusters were mainly distributed in the occipital, frontal, and temporal lobes. Compared with the NCs, decreased complexity was also found in the CUN.L in the MCI group (*P* = 0.08). These regions are mainly involved in short-term memory processing, visual recognition memory, rational thought processes and higher-level functions, basic visual processing, and motion perception (Goldman, 2013; de Schotten et al., 2014). The complexities of these brain regions in the patient groups decreased, and the brain function may also have been damaged. Liu et al. reported decreased complexities in some brain regions (i.e., the STG, ACG, CUN) in a complexity study of rs-fMRI signals in familial AD (Liu et al., 2013). Studies of EEG signals in AD and MCI patients also have reported results similar to ours. For example, Labate et al. (2012). measured dynamic EEG signal complexity in AD subjects using PE and found that the severity of disease was reflected in the dynamic complexity, and complexity reductions were present in the frontal and occipital areas (Labate et al., 2012). Timothy et al. found that in the frontal and temporal regions, the PEs of EEG recordings in an MCI group were significantly lower than those of controls (Timothy et al., 2014). The MMSE score has been demonstrated to be effective for the cognitive screening of the elderly and might help differentiate between AD and MCI, and the FAQ and CDR are frequently used indices of cognitive decline. Table 3 presents the positive correlations between of the PE with the MMSE in patients and the negative correlations of the PE with the FAQ and CDR. These findings indicated that lower MMSE and higher FAQ and CDR scores were observed in MCI and AD patient groups who exhibited lower complexity and cognitive decline.

These findings were supported by the decrease-in-complexity hypothesis of Lipsitz and Goldberger, which suggested that physiological diseases were associated with a generalized loss of complexity in the dynamics of healthy systems and hypothesizes that such a loss of complexity led to an impaired ability to adapt to physiological stress, that in turn results in functional loss and deficits (Lipsitz, 1992, 2004). These results indicated that AD patients demonstrated decreased complex behavioral output and suggested that AD patients had decreased brain complexity, resulting in cognitive decline.

### Potential explanations for the decreased complexity in AD

AD is characterized by the presence of neuritic plaques and neurofibrillary tangles and is accompanied by the loss of cortical neurons and synapses. These changes lead to cognitive and behavioral disturbances. Brain atrophy and the loss of cells are major changes in the AD brain. We examined the relationships between PE and the gray matter volume and glucose metabolism in patient groups. We found that the SOG exhibited a positive correlation between the PE and the gray matter volume, and two ROIs (i.e., the ITG and MOG) exhibited significant positive correlations between glucose metabolism and complexity. The SOG has been indicated in the gray matter atrophy in AD (Guo et al., 2010; Ouyang et al., 2015). Many studies have also found low cerebral glucose metabolism in some brain regions (i.e., the ITG and MOG) in AD and MCI (Castellano et al., 2015; Firbank et al., 2016). Because the related areas we found were few and not particularly significant, gray matter atrophy and decreased glucose metabolism may be indirect evidence for decreased complexity in AD.

A reliable explanation has been found for the decreased complexity of fMRI signals in AD. High regional functional homogeneity led to lower complexity. Functional homogeneity was measured by ReHo, which calculates the coherence of the BOLD signal in a given voxel with those of its nearest neighbors. In this study, we found significant negative correlations in the gray matter, white matter and some brain regions (i.e., the ITG, MFG, and MOG) between the PE and ReHo in the patient groups. He et al. investigated the pattern of regional coherence in AD patients using the ReHo index and found that the ReHo indices increased in the occipital (MOG and SOG) and temporal lobes (ITG), and significant negative correlations with the MMSE scores were presented in the MFG and CUN (He et al., 2007). These findings reflected the high homogeneity and low complexity in some brain areas in MCI and AD patients and may be helpful in the development of disease diagnoses.

### Limitations

There were several limitations to our research. A limitation of this study was that the nuisance covariates had influence on PE of fMRI signals. We found that differences between groups became smaller, after removing the effect of nuisance covariates. However, similar to the main result, significantly decreased complexities were found in frontal lobe in the AD group compared with the MCI groups and the NC group with weak statistical threshold (*P* < 0.01, uncorrected). According to these results, we speculated that the PE of fMRI signals might reflect the changes in complexity of brain activity and a fraction of nuisance signals. In addition, we also found the significant correlations between the PE and clinical measurements ReHo, gray matter volume, showing that the PE could reflect the abnormal brain activity of AD to a certain extent. The weak statistical threshold may be associated with the small sample size and unfavorable results after removing the nuisance signal. In the future research, we will take full account of the nuisance signals and improving the PE algorithm to measure the alterations in the complexity of brain activity more effectively.

Moreover, a limitation of the study was about the detailed information of subjects. Recent studies have demonstrated that age, sex, years of education, lifestyle, cardiovascular diseases, and risk factors (e.g., smoking and hypertension) were associated with cognitive decline and AD (Santos et al., 2014; Wirth et al., 2014). In this study, the data selected from the ADNI database, which did not publicly provide data about the risk factors related to AD. Another limitation was the number of time points of the fMRI data. According the PE algorithm, larger values of the embedding dimension contain more states of the reconstructed sequence. In this study, we chose m = 4, and there were 24 states. One hundred thirty time points might be inadequate for evaluating the abnormal complexity of fMRI signals in AD.

## Conclusions

Our analysis represents a novel implementation of temporal signal entropy (PE) to investigate the changes in the complexity of 4D fMRI brain signals in MCI and AD patients compared with healthy controls. We found decreased complexity in the AD group and found that the decreased complexity was significantly correlated with clinical measurements (i.e., the FAQ, MMSE, and CDR) in the patient groups. Furthermore, we also found a significant correlation between the PE and ReHo in the patient groups. These findings suggest that the complexity analysis of fMRI data using PE can provide important information about the fMRI characteristics of cognitively impaired conditions that can lead to AD. We suggest that PE is a useful and easily obtainable measure for identifying changes in AD brain dynamics. Future efforts will focus on increasing the fMRI database and applying the PE approach to other neurodegenerative diseases.

## Author contributions

BW and YN are co-first authors and completed the entire study of the experiment and writing. LM, RC, PY, HG, DL, and YG revised the manuscript. TY, JW, and HZ provided advice and guidance. JX provided the research ideas.

### Conflict of interest statement

The authors declare that the research was conducted in the absence of any commercial or financial relationships that could be construed as a potential conflict of interest.
