# Supplementary material for: Decreased Complexity in Alzheimer's Disease: Resting-State fMRI Evidence of Brain Entropy Mapping
Source: Front Aging Neurosci. 2017 Nov 20;9:378. doi: 10.3389/fnagi.2017.00378 (PMC5701971; doi:10.3389/fnagi.2017.00378)
Supplement: Supplementary file 1 [file Presentation1.pdf]

1. Data preprocessing

The preprocessing of rs-fMRI data was performed using the Data Processing Assistant for Resting-State fMRI (DPARSF) toolbox and the SPM8 package. Briefly, the preprocessing steps were as follows: the first 10 volumes of the functional images during the participant's adaptation to the circumstances were discarded; slice-timing correction was performed according to the last slice; the images were realigned for head movement compensation using a six-parameter rigid-body spatial transformation because excessive head motion may induce large artifacts in fMRI time series; the signal drift was removed using a linear model; the global signal, the motion parameters, the cerebrospinal fluid (CSF), and the white matter signals were removed as nuisance variables; the images were normalized to the Montreal Neurological Institute (MNI) space; finally, spatial smoothing of the brain PE maps was performed.

2. Statistical analyses

The first statistical tests were performed using the rs-fMRI Data Analysis Toolkit (REST 1.8). One-way ANOVA was performed to examine differences among the four groups (NC, EMCI, LMCI, and AD). Clusters that were significantly different after adjusting for age and sex differences were selected by setting $P<0.01$, uncorrected and cluster size>30.

The DPARSF toolbox was used to define the ROIs to extract the average PE, ReHo, and PDG-PET values according to the peak MNI coordinates (XYZ), and the radius of the spheres was 8 mm.

The subsequent statistical tests were performed using Statistical Package for Social Sciences (SPSS 20.0; New York, NY, USA) software. The averages PEs of the ROIs of each subject were obtained and one-way ANOVA was performed to examine the differences between the four groups. The relationships between the PE and the clinical measurements of MMSE, FAQ and CDR were analyzed using Pearson's correlations in the patient groups.

Pearson's correlation analyses of the PE with the ReHo and FDG-PET data were performed in the patient groups using SPSS. Moreover, we also performed correlation analyses between the PEs and the gray matter volumes in the patient groups.

3. Results

3.1 rs-fMRI PE brain maps

We extracted the mean PEs of the whole brain, gray matter (GM), white matter (WM), and cerebral spinal fluid (CSF). There were no differences in the whole brain

($F=0.436$, $P=0.728$) among the four groups. At the regional levels, 5 clusters were found to exhibit significant differences in PE among the four groups, as illustrated in Figure S1. The complexity differences among the four groups were mainly observed in the frontal lobes.

3.2 ROI analysis

We obtained 5 ROIs for the next analysis as presented in Table S1. Specifically, as presented in Table S1, the following regions exhibited significant differences:the left fusiform gyrus(FFG.L),the left rectus gyrus (REC.L), the inferior Frontal Gyrus (ORBinf.L, ORBinf.R), the right middle frontal Gyrus(MFG.R). The results of one-way ANOVAs revealed significant effects of group in 5 brain regions that exhibited significantly decreased complexity in the AD group compared with the MCI groups and the NC group (Figure S2).

3.3 Relationships between PE and clinical measurements

First, we performed correlation analyses of the MMSE scores with the mean PEs of the whole brain in the patient groups (EMCI+LMCI+AD) and found that no correlations. We also examined the correlations of the MMSE scores with the PEs of the 5 ROIs in the pooled patient groups (EMCI+LMCI+AD). The results were presented in Table S2. 4 ROIs exhibited positive correlations between the PEs and MMSE scores ($r>0.189$, $P<0.076$). A higher MMSE score indicates higher cognitive ability. There were no correlations of the FAQ scores with the mean PEs of the whole brain in patient groups. The PEs of 5 ROIs exhibited strong negative correlations with the FAQ scores ($r<-0.236$, $P<0.026$).A higher FAQ score indicates poorer functional performance. There were no correlations of the CDR scores with the mean PE of the whole brain in the patient groups. The PEs of 5 ROIs exhibited strong negative correlations with the CDR scores ($r<-0.238$, $P<0.025$). A higher CDR score indicates the presence of dementia. The correlation analyses were performed between the PE and the clinical measurements in the four groups (Table S3). Consistent significant correlations were found.

3.4 Relationships between PE and ReHo

We extracted the ReHos of 5 ROIs according to the peak MNI coordinates (Table S1), and the sphere radius was 8 mm. We explored the relationship between PE and ReHo in the pooled groups (EMCI+LMCI+AD). The results were presented in Table S4. 3 ROIs (FFG.L, ORBinf.L, and MFG.R) exhibited significant negative correlations between the PE and ReHo in the patient groups. The results illustrated that high regional spontaneous activities may be associated with a decrease in complexity.

Correlation analyses in the four groups (NC+EMCI+LMCI+AD) were also

performed between the PE and ReHo (Table S5). Consistent significant correlations were found.

3.5 Relationships between PE and the gray matter volume, FDG-PET

We extracted the gray matter volumes of 5 ROIs according to the peak MNI coordinates, and the sphere radius was 8 mm. Then, we explored the relationships between the PEs and the gray matter volumes in the patient groups. The FFG.L(r=0.024, P=0.019) and MFG.R(r=0.270, P=0.008) exhibited a positive correlation between the PE and the gray matter volume in the patient groups (Table S4). Correlation analyses in the four groups (NC+EMCI+LMCI+AD) were also performed between the PE and gray matter volume, and the MFG.R exhibited a significant positive correlation (r=0.185, P=0.041) between the PE and the gray matter volume (Table S5).

Finally, the FDG-PET data of the 5 ROIs from the same group of subjects were extracted. Pearson's correlation analyses of the PE and FDG-PET were performed and no correlations were found in the patient groups (Table S4) and in the four groups (Table S5).

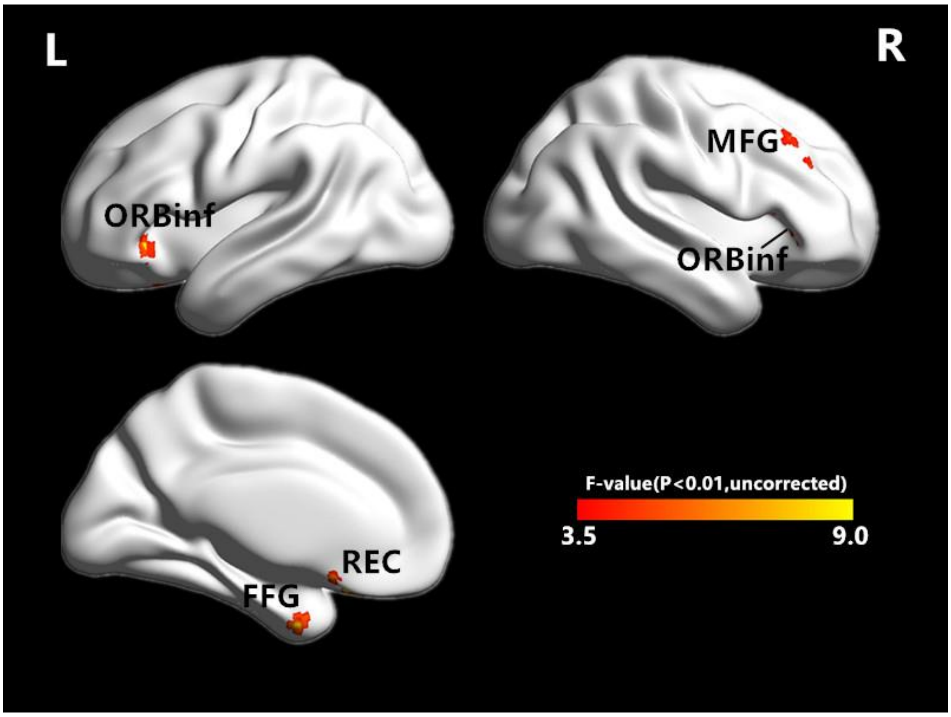

Figure S1. Surface-rendered images showed the differences between the control and patient groups after adjusting for age and sex. The regions showed exhibited different complexities among the four groups (threshold P<0.01, uncorrected). See Table S1 for a complete list of these regions.

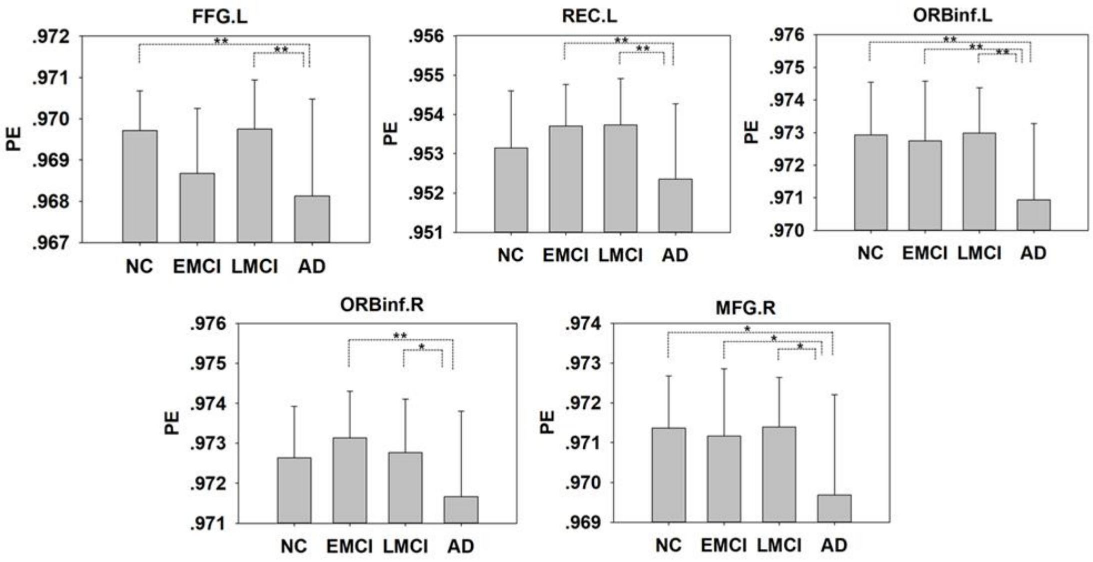

Figure S2. The PE values of the NC, EMCI, LMCI and AD subjects. Significant differences between pairs of groups are indicated (P<0.05, uncorrected). * P<0.05, ** P<0.01. The error bars indicate the SDs.

Table S1. Characteristics of the brain regions that were significantly different among the four groups

Brain Region	AAL.Abbr	MNI (X, Y, Z)	Cluster voxels	Voxel F value
Fusiform Gyrus	FFG.L	(-27, 0, -36)	32	8.19
Rectus Gyrus	REC.L	(-15, 21, -21)	43	8.64
Inferior Frontal Gyrus	ORBinf.L	(-39, 30, -6)	44	8.95
Inferior Frontal Gyrus	ORBinf.R	(39, 33, -3)	36	6.29
Middle Frontal Gyrus	MFG.R	(39, 36, 27)	40	5.74

The location coordinates are those of the peak significance in each region (P<0.01, uncorrected).

Table S2. Results of the correlation analyses between the PE maps and the MMSE, FAQ, and CDR scores in the patient groups (EMCI+LMCI+AD).

Brain region Abbr.	MMSE (r, P)	FAQ (r, P)	CDR(r, P)
FFG.L	0.164, 0.125	-0.294, 0.005**	-0.356, 0.001***
REC.L	0.198, 0.062	-0.285, 0.007**	-0.271, 0.010**
ORBinf.L	0.189, 0.076	-0.293, 0.005**	-0.263, 0.013*
ORBinf.R	0.228, 0.032*	-0.236, 0.026*	-0.238, 0.025*
MFG.R	0.190, 0.074	-0.278, 0.008**	-0.300, 0.004**

In the table, r is the Pearson correlation coefficient, and P indicates the level of statistical significance. * P<0.05, ** P<0.01, *** P<0.001.

Table S3. Results of the correlation analyses between the PE maps and the MMSE, FAQ, and CDR scores in the four groups (NC+EMCI+LMCI+AD).

Brain region Abbr.	MMSE (r, P)	FAQ (r, P)	CDR(r, P)
FFG.L	0.223, 0.016*	-0.337, <0.001***	-0.366, <0.001***
REC.L	0.146, 0.119	-0.223, 0.016*	-0.122, 0.192
ORBinf.L	0.194, 0.037**	-0.292, 0.001***	-0.232, 0.012*
ORBinf.R	0.168, 0.071	-0.196, 0.035*	-0.113, 0.225
MFG.R	0.192, 0.039*	-0.283, 0.002**	-0.252, 0.006**

In the table, r is the Pearson correlation coefficient, and P indicates the level of statistical significance. * P<0.05, ** P<0.01, *** P<0.001.

Table S4. Results of the correlation analyses between the PE maps and the ReHo, gray matter volume and FDG-PET values in the patient groups (EMCI+LMCI+AD).

Brain region Abbr.	ReHo (r, P)	GMV (r, P)	FDG-PET(r, P)
FFG.L	-0.217, 0.035*	0.241, 0.019*	0.066, 0.582
REC.L	-0.000,0.952	0.095, 0.364	0.075, 0.526
ORBinf.L	-0.237, 0.016*	0.159, 0.125	0.119, 0.316
ORBinf.R	-0.144, 0.166	0.053, 0.609	0.100, 0.398
MFG.R	-0.162, 0.087	0.270, 0.008**	0.112, 0.344

In the table, r is the Pearson correlation coefficient, and P indicates the level of statistical significance. *P<0.05, **P<0.01. GMV, Gray Matter Volume.

Table S5. Results of the correlation analyses between the PE maps and the ReHo, gray matter volume and FDG-PET values in the four groups (NC+EMCI+LMCI+AD).

Brain region Abbr.	ReHo (r, P)	GMV (r, P)	FDG-PET(r, P)
FFG.L	-0.195, 0.030*	0.124, 0.172	0.024, 0.823
REC.L	-0.015,0.872	0.135, 0.136	0.014, 0.896
ORBinf.L	-0.216, 0.007**	0.049, 0.594	0.070, 0.505
ORBinf.R	-0.095, 0.294	0.130, 0.150	0.055, 0.601
MFG.R	-0.176, 0.050*	0.185, 0.041*	0.075, 0.476

In the table, r is the Pearson correlation coefficient, and P indicates the level of statistical significance. * P<0.05, ** P<0.01. GMV, Gray Matter Volume.
