# Supplementary material for: Decreased Complexity in Alzheimer's Disease: Resting-State fMRI Evidence of Brain Entropy Mapping
Source: Front Aging Neurosci. 2017 Nov 20;9:378. doi: 10.3389/fnagi.2017.00378 (PMC5701971; doi:10.3389/fnagi.2017.00378)
Supplement: Supplementary file 2 [file Table1.docx]

**Table S1** Results of the correlation analyses between the PE maps and the MMSE, FAQ, and CDR scores in the four groups (NC+EMCI+LMCI+AD).

| Brain region Abbr. | MMSE (r, P) | FAQ (r, P) | CDR(r, P) |
| --- | --- | --- | --- |
| ITG.R | 0.270, 0.003** | -0.200, 0.031* | -0.217,0.019* |
| MFG.R | 0.354, <0.001*** | -0.260, 0.005** | -0.292,0.001*** |
| SFGdor.L | 0.274, 0.003** | -0.325, <0.001*** | -0.337,<0.001*** |
| ACG.L | 0.279, 0.002** | -0.213, 0.022* | -0.317,0.001*** |
| CUN.R | 0.329, <0.001*** | -0.227, 0.014* | -0.170,0.068 |
| CUN.L | 0.233, 0.012* | -0.175, 0.060 | -0.179,0.054 |
| MOG.R | 0.357, <0.001 *** | -0.296,<0.001*** | -0.292,0.001*** |
| SOG.R | 0.308, 0.001*** | -0.277, 0.003** | -0.205,0.027* |
| GM | 0.076, 0.417 | -0.041,0.664 | -0.030,0.747 |
| WM | 0.191,0.041* | -0.116,0.215 | -0.117,0.209 |

In the table, r is the Pearson correlation coefficient, and P indicates the level of statistical significance. * P<0.05, ** P<0.01, *** P<0.001. GM, Gray Matter; WM, White Matter.
