# Supplementary material for: Decreased Complexity in Alzheimer's Disease: Resting-State fMRI Evidence of Brain Entropy Mapping
Source: Front Aging Neurosci. 2017 Nov 20;9:378. doi: 10.3389/fnagi.2017.00378 (PMC5701971; doi:10.3389/fnagi.2017.00378)
Supplement: Supplementary file 3 [file Table2.docx]

**Table S2** Results of the correlation analyses between the PE maps and the ReHo, gray matter volume and FDG-PET values in four groups (NC+EMCI+LMCI+AD).

| Brain region Abbr. | ReHo (r, P) | GMV (r, P) | FDG-PET(r, P) |
| --- | --- | --- | --- |
| ITG.R | -0.351,<0.001*** | -0.026,0.775 | 0.207,0.047* |
| MFG.R | -0.317,<0.001*** | -0.002,0.982 | 0.023,0.824 |
| SFGdor.L | -0.196,0.030* | -0.035,0.701 | 0.070,0.507 |
| ACG.L | -0.011,0.900 | 0.052,0.567 | 0.136,0.195 |
| CUN.R | -0.013,0.884 | 0.023,0.803 | 0.024,0.819 |
| CUN.L | -0.025,0.786 | 0.009,0.919 | 0.039,0.711 |
| MOG.R | -0.144,0.111 | 0.052,0.567 | 0.294,0.004** |
| SOG.R | -0.062,0.499 | 0.230,0.010** | 0.129,0.216 |
| GM | -0.276,0.002** | 0.068,0.519 | 0.020,0.847 |
| WM | -0.393,<0.001*** | --- | 0.007,0.948 |

In the table, r is the Pearson correlation coefficient, and P indicates the level of statistical significance. *P<0.05, **P<0.01, *** P<0.001. GMV, Gray Matter Volume; GM, Gray Matter; WM, White Matter.
